# IC100, a humanized therapeutic monoclonal anti-ASC antibody alleviates oxygen-induced retinopathy in mice

**DOI:** 10.1007/s10456-024-09917-9

**Published:** 2024-05-06

**Authors:** Huijun Yuan, Shaoyi Chen, Matthew R. Duncan, Juan Pablo de Rivero Vaccari, Robert W. Keane, W. Dalton Dietrich, Tsung-Han Chou, Merline Benny, Augusto F. Schmidt, Karen Young, Kevin K. Park, Vittorio Porciatti, M. Elizabeth Hartnett, Shu Wu

**Affiliations:** 1 Department of Pediatrics/Division of Neonatology, Batchelor Children’s Research Institute and Holtz Children’s Hospital, University of Miami Miller School of Medicine, P. O. Box 016960, Miami, FL 33101 USA; 2 The Miami Project to Cure Paralysis and Department of Neurological Surgery, University of Miami Miller School of Medicine, Miami, FL USA; 3 Department of Physiology and Biophysics, University of Miami Miller School of Medicine, Miami, FL USA; 4 Bascom Palmer Eye Institute, University of Miami Miller School of Medicine, Miami, FL USA; 5 Department of Ophthalmology, University of Texas Southwestern Medical Center, Dallas, TX USA; 6 Byers Eye Institute at Stanford University, Palo Alto, CA USA

**Keywords:** ASC, Oxygen-induced retinopathy, IC100, Microglia, Transcriptome

## Abstract

**Background:**

Retinopathy of prematurity (ROP), which often presents with bronchopulmonary dysplasia (BPD), is among the most common morbidities affecting extremely premature infants and is a leading cause of severe vision impairment in children worldwide. Activations of the inflammasome cascade and microglia have been implicated in playing a role in the development of both ROP and BPD. Apoptosis-associated speck-like protein containing a caspase recruitment domain (ASC) is pivotal in inflammasome assembly. Utilizing mouse models of both oxygen-induced retinopathy (OIR) and BPD, this study was designed to test the hypothesis that hyperoxia induces ASC speck formation, which leads to microglial activation and retinopathy, and that inhibition of ASC speck formation by a humanized monoclonal antibody, IC100, directed against ASC, will ameliorate microglial activation and abnormal retinal vascular formation.

**Methods:**

We first tested ASC speck formation in the retina of ASC-citrine reporter mice expressing ASC fusion protein with a C-terminal citrine (fluorescent GFP isoform) using a BPD model that causes both lung and eye injury by exposing newborn mice to room air (RA) or 85% O_2_ from postnatal day (P) 1 to P14. The retinas were dissected on P14 and retinal flat mounts were used to detect vascular endothelium with AF-594-conjugated isolectin B4 (IB4) and citrine-tagged ASC specks. To assess the effects of IC100 on an OIR model, newborn ASC citrine reporter mice and wildtype mice (C57BL/6 J) were exposed to RA from P1 to P6, then 75% O_2_ from P7 to P11, and then to RA from P12 to P18. At P12 mice were randomized to the following groups: RA with placebo PBS (RA-PBS), O_2_ with PBS (O_2_-PBS), O_2_ + IC100 intravitreal injection (O_2_-IC100-IVT), and O_2_ + IC100 intraperitoneal injection (O_2_-IC100-IP). Retinal vascularization was evaluated by flat mount staining with IB4. Microglial activation was detected by immunofluorescence staining for allograft inflammatory factor 1 (AIF-1) and CD206. Retinal structure was analyzed on H&E-stained sections, and function was analyzed by pattern electroretinography (PERG). RNA-sequencing (RNA-seq) of the retinas was performed to determine the transcriptional effects of IC100 treatment in OIR.

**Results:**

ASC specks were significantly increased in the retinas by hyperoxia exposure and colocalized with the abnormal vasculature in both BPD and OIR models, and this was associated with increased microglial activation. Treatment with IC100-IVT or IC100-IP significantly reduced vaso-obliteration and intravitreal neovascularization. IC100-IVT treatment also reduced retinal microglial activation, restored retinal structure, and improved retinal function. RNA-seq showed that IC100 treatment corrected the induction of genes associated with angiogenesis, leukocyte migration, and VEGF signaling caused by O_2_. IC100 also corrected the suppression of genes associated with cell junction assembly, neuron projection, and neuron recognition caused by O_2_.

**Conclusion:**

These data demonstrate the crucial role of ASC in the pathogenesis of OIR and the efficacy of a humanized therapeutic anti-ASC antibody in treating OIR mice. Thus, this anti-ASC antibody may potentially be considered in diseases associated with oxygen stresses and retinopathy, such as ROP.

**Supplementary Information:**

The online version contains supplementary material available at 10.1007/s10456-024-09917-9.

## Introduction

Retinopathy of prematurity (ROP), which often presents with bronchopulmonary dysplasia (BPD), is among the most common morbidities, affecting approximately 60% of very low-birthweight infants needing oxygen therapy [1, 2]. ROP is the leading cause of childhood vision impairment and blindness worldwide [3–5]. The pathological hallmarks of ROP are characterized in 2 phases, with delayed vascular development in phase 1 and intravitreal neovascularization in phase 2 [1, 2]. Laser and anti-VEGF therapy are current treatments for severe ROP [6, 7]. Laser therapy can reduce intravitreal neovascularization and decrease the possibility of progression to retinal detachment. However, some reported side effects of laser therapy include induced myopic refractive error and visual field loss [6, 8]. Anti-VEGF therapy is considered in some forms of severe ROP, but VEGF also plays critical physiological functions in vital organ development, such as in the lungs and brains of these infants [9, 10]. It has been reported that anti-VEGF therapy for ROP may have side effects on system vascular development and adverse effects on brain and lung development [9, 11, 12]. Thus, new effective, preventive, and treatment strategies with fewer side effects are needed.

The mouse model of oxygen-induced retinopathy (OIR) is useful for testing the effects of high oxygen exposure on both the initial developing neonatal retinal vasculature and subsequent intravitreal neovascularization. First high oxygen damages newly developed capillaries and then following the return to room air (RA), the avascular retina stimulates the development of intravitreal tufts. Recent studies report that neurons, astrocytes, microglia inflammation, and pyroptosis contribute to vasculopathy due to elevated reactive oxygen species (ROS) and inflammation in OIR models [13–15]. Most recently, researchers reported that retinal microglia play important roles in the protection of vascular damage in a mouse model of OIR [16].

Inflammasomes are multi-protein complexes that mediate proteolytic cleavage of gasdermin D (GSDMD), pro-IL-1β, and pro-IL-18 by caspase-1 [17–19]. Apoptosis-associated speck-like protein containing a caspase recruitment domain (ASC) is pivotal in inflammasome assembly and activation of caspase-1 [20, 21]. A hallmark of inflammasome activation is ASC assembly into large oligomer protein complexes, called ASC specks (~ 1–2 µm). Macromolecular ASC speck formation by oligomerization of pyrin domains (PYD) creates a multitude of activation sites for caspase-1. Activated caspase-1 cleaves GSDMD to release a 30-kDa N-terminal domain, GSDMD-p30, which oligomerizes in the cell membrane to form pores that cause pyroptosis [22, 23]. In addition, the GSDMD pores allow rapid release of active IL-1β and IL-18, resulting in secondary inflammation [24]. Moreover, inhibition of the NLRP3 inflammasome with MCC950 suppresses the development of retinal neovascularization in OIR mice [25]. We have reported that GSDMD gene knockout prevents both vaso-obliteration and intravitreal neovascularization in OIR mice [26]. However, there are no reports on the effects of ASC inhibitory strategies in treating ROP or OIR mice.

IC100 is a humanized IgG4 monoclonal antibody that targets ASC, disrupts ASC oligomerization and speck formation, and inhibits IL-1β release [27]. It has been reported that IC100 suppresses the immune-inflammatory response in experimental autoimmune encephalomyelitis models of multiple sclerosis and traumatic brain injury [27, 28].

In this study, we tested the hypothesis that hyperoxia induces ASC speck formation, which leads to microglial activation and retinopathy, and that inhibition of ASC speck formation by IC100 will ameliorate microglial activation and abnormal retinal vascular formation. To test these hypotheses, we used the mouse OIR and BPD models and provided insights into the mechanistic functions of ASC in the pathogenesis of OIR and the utility of ASC inhibition.

## Materials and methods

### Material

Please see the online supplement for material lists.

IC100 (IgG4) was developed by humanization of a mouse monoclonal (IgG1) against human ASC (Abzena, Cambridge England). It was cloned into a CHO cell manufacturing cell line (Selexis, Geneva, Switzerland) and purified from CHO cell supernatants using ProSepA high-capacity column chromatography (Antibody Solutions, Santa Clara, CA) [27, 29].

### Animals and study approval

The Animal Care and Use Committee of the University of Miami Miller School of Medicine approved the experimental protocol. All animals were cared for according to the National Institutes of Health guidelines for the use and care of animals. Wildtype mice (C57BL/6 J) and R26-CAG-ASC-citrine mice with a fluorescent reporter of inflammasome complex activation, which enables the visualization of ASC specks by fluorescent microscopy [30], were purchased from the Jackson Laboratory. The study is reported in accordance with the ARRIVE guidelines.

### BPD model in hyperoxia-exposed ASC-citrine mice

To test ASC activation in the retina, ASC-citrine reporter mice were exposed to 85% oxygen (O_2_) from postnatal day (P) 1 to P14 to induce multi-organ injury, including eye, brain, and lung which commonly co-exist in mice and extremely preterm infants [26, 29]. Nursing dams were rotated between room air (RA) to 85% O_2_ every other day. The retinas were dissected on P14, and retinal vessels were stained with AF-594-conjugated isolectin B4 (IB4) to assess vasculature. The inflammasome complex activation was detected by ASC speck formation in the retinas. Images of retinal flat mount were captured by fluorescent confocal microscopy.

### OIR model and IC100 treatment

The OIR mouse model was generated in ASC-citrine reporter mice and C57BL/6 J wildtype mice as previously described [30] by exposing newborn mice to RA from P1 to P6, then to 75% O_2_ from P7 to P11, and then to RA from P12 to P18. Control mice were exposed to RA from P1 to P18. To test the effects of IC100, the RA-exposed and oxygen-exposed mice were randomized on P12 to the following groups: RA + placebo PBS (RA-PBS), RA + IC100 intravitreal (RA-IC100-IVT) injection, 75% O_2_ with PBS (O_2_-PBS), 75% O_2_ + IC100-IVT, and 75% O_2_ + IC100 intraperitoneal (O_2_-IC100-IP) injection. For IVT injection, 1 dose of IC100 at 2.5 µg/0.5µL/eye was injected into both eyes of mice on P12. For IP injection, IC100 at a dose of 10 µg/g or 20 µg/g mouse body weight was administered on P12, P14, and P16. Some of these mice were sacrificed for retinal dissection on P18 and others were used for pattern electroretinogram (PERG) analysis on P30.

### Retinal whole mount, section immunofluorescent staining and vascular analysis

Upon sacrifice, the eyeballs were enucleated and fixed in 4% paraformaldehyde/PBS for 45 min at room temperature. Retinas were dissected and permeabilized in 0.5% Triton X100/PBS overnight at 4 °C. After blocking with 10% donkey serum in PBS for 1 h at room temperature, the retinal whole mounts were stained with IB4 for retinal vessel labeling and with a Brilliant Violet 421™-labeled anti-CD206 antibody, which is a marker for alternative microglial activation (M2 microglia) [33] and allograft inflammatory factor 1 (AIF-1) that is a marker for activated microglial cells [34] overnight at 4 °C. The retinal whole mounts were washed with PBS three times. AIF-1 was probed using an AF-488-labeled donkey anti-goat antibody for 1 h at room temperature. After three washes with PBS, the retinas were flat mounted with 50% glycerol. The retinal flat mount images were taken and analyzed by fluorescent microscopy (Zeiss). Vaso-obliteration, as avascular retina, and intravitreal neovascularization and related tufts were quantified by image J or Zeiss Software as previously described [26]. For retinal cross-section analysis, fixed eyeballs were placed in 10%–30% sucrose for 20 min before the eyeballs were embedded in OCT. Eight µm retinal sections were cut, blocked with animal-free Blocker® for 1 h, and incubated with primary antibodies overnight at 4 °C and secondary antibody for 1 h. The retinal sections were mounted with antifade mounting medium with 4′,6-diamidino-2-phenylindole (DAPI) and analyzed by the Dragonfly high speed confocal microscopy.

### Western blot analysis

The vitreous fluid was collected from 5 RA-exposed and 5 OIR wildtype mice with PBS-containing protein inhibitor cocktails, and they were pooled in each group, centrifuged at 2000 rpm for 10 min at 4 °C. The protein concentration of the supernatant was determined by BSA protein assay, and 15 µg of proteins from RA and OIR vitreous fluid were separated by 4–20% SDS-page gel (Bio Rad, Cat. 4568093). Proteins were then transferred to immobilon-P polyvinylidene membrane. The membrane was blocked with 3% BSA for 1 h and probed with an ASC antibody (Santa Cruz, Cat. sc-515414) overnight at 4 °C. After washing 3 times with 0.1% Tween-20 PBS, the membrane was incubated with horseradish peroxidase-conjugated secondary antibody for 1 h. The antibody bound proteins were detected by ECL chemiluminescence and visualized with ChemiDoc™ MP Imager (Bio-Rad). The membrane was then stripped with 0.2 N NaOH and reincubated with a primary antibody reactive with a normalization protein, β-actin.

### Histopathology analysis

Flat mount retinas were cryoprotected in 10% to 30% sucrose before embedding in optimal cutting temperature (OCT) after they were analyzed as whole mounts and 8 μm sections were prepared. H&E staining was performed, and images were taken by bright filed light microscopy (Zeiss, Germany).

### PERG analysis

PERG was used to measure the function of the retina by recording the amplitudes and latency of our mice at P30 [31]. Mice were weighed and anesthetized by IP injection of a mixture of ketamine (80 mg/kg body weight) and xylazine (10 mg/kg body weight). Mice were then placed on a feedback-controlled heating pad to maintain body temperature at 37.6 °C. Mice were positioned 20 cm from the stimulus monitor with their head angle tilted at 45 degrees to provide direct exposure of the grating stimulus to the recorded eyes. PERG signals were recorded from a common subcutaneous needle (Grass Technologies, West Warwick, RI, USA) placed in the snout and referenced to a similar electrode placed in the back of the head. A subcutaneous electrode placed at the root of the tail served as ground. Pupils were natural without dilation. A small drop of balanced saline solution was applied topically as necessary to prevent corneal dryness. Visual stimuli consisting of horizontal gratings (95% contrast, 0.06 cycles/degree spatial frequency), 700 cd/m2 mean luminance were generated on two (15 cm × 15 cm) LED tablet displays (Jorvec Corp, Miami, FL, USA) and presented at each eye separately at a distance of 10 cm. Gratings reverse in contrast at slightly different temporal frequency (OD, 0.984 Hz; OS, 0.992 Hz) to allow deconvolution of the signal and retrieval of PERG from each eye. PERG signals were fed to an Opti-Amp bioamplifier (Intelligent Hearing Systems Inc., Miami, FL, USA) amplified (10,000-fold), filtered (1–300 Hz, 6 dB/oct), and averaged (OD, 372 epochs of 492 ms; OS, 372 epochs of 496 ms) using a Universal Smart Box acquisition system (Intelligent Hearing Systems Inc., Miami, FL, USA). Three consecutive responses were recorded and superimposed to check for consistency and then averaged.

### RNA isolation, qRT-PCR, and RNA sequencing

Total RNA from retinas was extracted with Trizol, and cDNA was synthesized with an iScript cDNA synthesis Kit. Gene expression levels for Il1b, Il6, Il18, tumor necrosis factor (Tnf), Pycard (Asc), Nlrp3, Gsdmd, and Vegf were determined by qRT-PCR using Taqman gene expression Assays and a Bio-Rad Cycler System. The primers for these genes were obtained from ThermoFisher Scientific. The relative gene expression levels were normalized to 18S RNA. RNA sequencing (RNA-seq) was performed by Novogene (Sacramento, CA) using Illumina NovaSeq platforms with a read depth of 30 million reads per sample for 150 bp paired-end reads, as we have previously described [26]. The raw sequence reads in FASTQ format were aligned to the mouse (*Mus musculus*) genome build mm_GRCm39_104 using Kallisto, followed by gene summarization with tximport. After checking data quality, differential expression analyses comparing treatment groups to control and each other were performed using DESeq2 with false discovery adjustment. Genes were considered differentially expressed based on their fold-change relative to control (= or > 1.5), *P*-value (< 0.05), and FDR (< 0.1). Lists of differentially expressed genes were used for functional enrichment analysis of Gene Ontology terms and pathways using the clusterProfiler package (PMID 34557778). Only unique terms associated with either induced or suppressed genes and at least 2 genes were reported.

### Statistical analysis

All the experiments were performed and analyzed blindly. ANOVA followed by Turkey’s post-hoc analyses were done by Prism v9.0, Graphpad Software Inc. (San Diego, CA). Data were presented as Mean ± SEM, and significance was set at* P* < 0.05.

## Results

### ASC specks are formed in the retinas of hyperoxia-induced BPD and OIR models

**Fig. 1:**
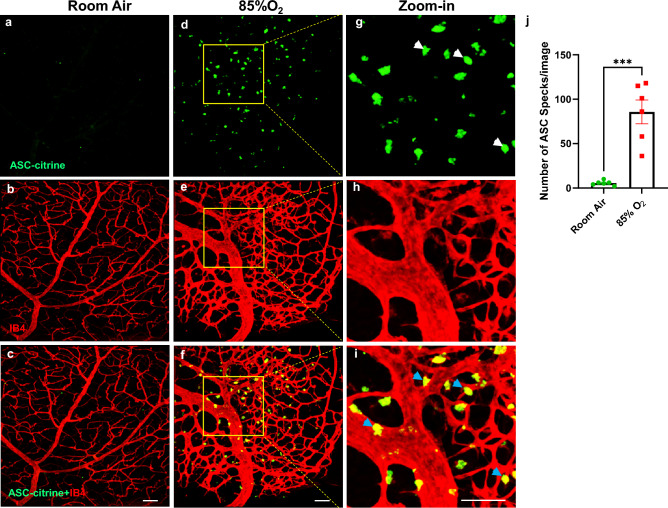
Immunofluorescent confocal microscopy images of BPD model ASC-citrine mouse whole mount retinas. **a**–**c** Images of RA-exposed (RA) mouse whole mount retinas: **a** Green, ASC-citrine. **b** Normal vasculature, red (IB4 staining) **c** Merged image. **d**–**f** Images of 85% O_2_-exposed mouse whole mount retinas: **d** Green, ASC-citrine specks. **e** Disorganized proliferative vasculature, red (IB4 staining) **f** Merged image of **d & e**.** g**–**i** Zoom-in images of **d**–**f**. **g** ASC-citrine specks (white arrow). **h** Proliferative vasculature, red (IB4 staining). **i** The merged image show ASC-citrine specks colocalized with abnormal vasculature (yellow, blue arrow). **j** Quantification of ASC-citrine speck images. ***P < 0.0001. n = 6 mice/group. scale bars: 50 µm

ASC speck formation is widely used as a readout for inflammasome activation [32–35]. To determine if ASC specks formed in response to hyperoxia challenge in a BPD model, we used ASC-citrine reporter mice (ASC-citrine) [32] and exposed them to RA or 85% O_2_ for 2 weeks from P1 to P14. Our previous studies have shown that this level of oxygen-induced lung injury similar to BPD [26] and brain injury [36]. The ASC specks were induced in the lungs and brains of ASC-citrine reporter mice (data not shown). We performed retinal flat mount staining for blood vessels with IB4. Confocal fluorescence microscope images showed that ASC-citrine fusion protein formed numerous large green specks in hyperoxia-exposed retinas, indicating that the ASC-citrine fusion protein was incorporated into an oligomerizing assembled inflammasome complex (Fig. 1d & g**,** green, white arrow). These ASC specks were colocalized with disorganized and proliferative vasculature stained by IB4 (Fig. 1f & i, yellow, blue arrow). The number of ASC specks in hyperoxia-exposed retinas was more than tenfold higher than in RA controls (Fig. 1j). This finding indicates that hyperoxia activates the inflammasome pathway, which is highly correlated with abnormal proliferative angiogenesis.

**Fig. 2:**
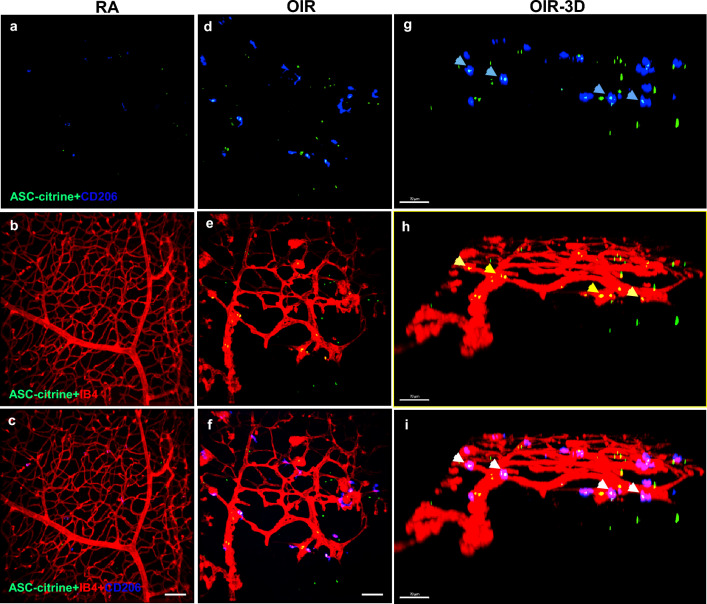
Immunofluorescent confocal microscopy images of OIR model ASC-citrine mouse retinal flat mount staining. **a**–**c** Images of RA mouse whole mount retinas: **a** Merged image of ASC-citrine (green) and CD206 positive microglia (blue). **b** Merged image of normal vasculature (red, IB4 staining) and ASC-citrine. **c** Merged image of ASC-citrine, blood vessel and microglia. **d**–**f** Images of OIR mouse retinal flat mount staining: **d** Merged image of ASC-citrine specks (Green) and CD206 positive microglia (blue). **e** Merged image of neovascular tufts (red, IB4 staining) and ASC-citrine specks (green). **f** Merged image of OIR retinal neovascular tufts, ASC-citrine specks and CD206 positive microglia. **g**–**i** 3D Images of images **d**–**f** scale bars: 70 µm

We next evaluated ASC speck formation in an OIR model in ASC-citrine reporter mice and wildtype C57BL/6 J mise [30]. In this model, the hyperoxia exposure induces vascular dysplasia of the central retina, the retinas became hypoxic, and the expression of angiogenic factors is upregulated, leading to retinal neovascular growth during RA recovery [26]. Firstly, we examined ASC speck formation in OIR, ASC-citrine mice in retinal flat mounts by confocal microscopy for citrine positive specks and microglial cells detected by staining for CD206. The OIR mice had increased ASC specks and CD206-labeled microglia (Fig. 2d) in the neovascular areas (Fig. 2e & 2f). This is better appreciated in the 3D immunofluorescent confocal images which show that ASC specks were highly activated and colocalized with CD206-labeled microglia **(**Fig. 2g) in the neovascular tuft areas (Fig. 2h & 2i). Thus, ASC speck formation is highly correlated with alternatively activated microglia, which may play a role in promoting intravitreal neovascularization.

**Fig. 3:**
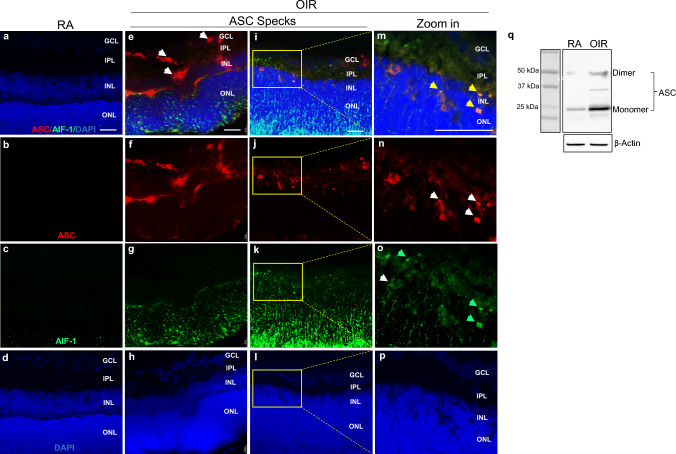
ASC speck formation in wildtype OIR model mouse retinas. Immunofluorescent microscopy images of mouse retinal cross-sections.** a**–**d** Representative immunofluorescent microscopy images of RA mouse retinal sections.** e**–**l** Representative immunofluorescent microscopy images of OIR mouse retinal sections. **m**–**p** Zoom-in images of** i**–**l**. ASC specks were detected in the intracellular in ganglion cell layer (GCL, **e & i,** white arrow), extracellular in inner plexiform layer (IPL, **e & i**, white arrow), and activated microglia cells in inner nuclear layer (INL, **k & o**, green arrow) in OIR mouse retinas. ASC specks, in the intracellular and/or extracellular matrix, (**m,** yellow arrow) colocalized with activated microglia (**o**, green arrow) in OIR mouse retinas. n = 5 mice/group. Scale bar: 25 µM. **q** Representative images of Western blot analysis of ASC from pooled 5 RA and 5 OIR mouse vitreous fluids

We then examined ASC expression in OIR wildtype mice by immunofluorescence staining with an anti-ASC antibody and found that ASC specks were present in the ganglion cell layer (GCL), inner plexiform layer (IPL), and inner nuclear layer (INL) (Fig. 3e**,** red, white arrow) or released extracellularly near the IPL and INL. Activated microglial cells stained by AIF-1 were also detected in the INL and IPL (Fig. 3g, k & o**,** green arrow). Therefore, microglia as resident immune cells in the retina were highly activated and colocalized with ASC specks in OIR mouse retinas (Fig. 3m**,** yellow arrow). Additionally, we tested if ASC specks were released into the vitreous fluid in OIR mice. Western blot showed that the intensity of the bands of ASC monomers and dimers from OIR mouse vitreous fluid was increased compared to age-matched RA mice (Fig. 3q). Taken together, our findings reveal that hyperoxia exposure induces ASC speck activation in the retina that is released into ocular vitreous fluid in wildtype mice.

### IC100 alleviates pathological vaso-obliteration and intravitreal neovascularization in OIR mice

**Fig. 4:**
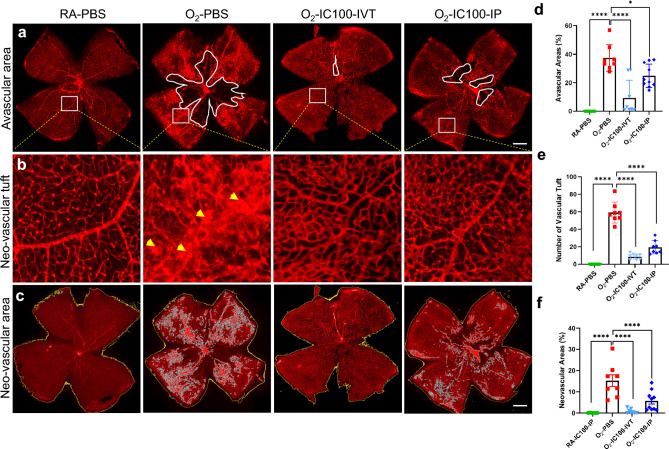
Anti-ASC antibody, IC100 reduced oxygen-induced pathological vaso-obliteration and intravitreal neovascularization. **a** Representative images of P18 mouse retinal flat mounts; control: RA + placebo PBS (RA-PBS), OIR: OIR treated with PBS (O_2_-PBS) by IVT injection, IC100 by IVT (O_2_-IC100-IVT), and IC100 by IP (O_2_-IC100-IP) injection. The avascular areas were circled in white. **b** The white boxes in **a** were enlarged eightfold, and the yellow arrows indicate vascular tufts. **c** Intravitreal neovascularization was highlighted by white dots generated by Zeiss microscope software based on vascular intensity. **d** Quantifications of avascular areas. The O_2_-PBS group had increased avascular areas compared to the RA-PBS group (****P < 0.0001). But treatment with IC100 by IVT and IP injection significantly reduced the avascular areas compared to the O_2_-PBS group (O_2_-IC100-IVT, ****P < 0.0001, O_2_-IC100-IP, *P < 0.05). **e** Measurements of neovascular tufts. The O_2_-PBS group had increased vascular tufts compared to the RA-PBS group (****P < 0.0001). But treatment with IC100 by IVT and IP injection significantly reduced the neovascular turfs compared to the O_2_-PBS group (****P < 0.0001). **f** Assessment of intravitreal neovascularization. The O_2_-PBS group had increased intravitreal neovascularization compared to the RA-PBS group (****P < 0.0001). But treatment with IC100 by IVT and IP injection significantly reduced the neovascular areas compared to the O_2_-PBS group (****P < 0.0001). RA-PBS, O_2_-PBS, and O_2_-IC100-IVT groups: n = 4 mice/group. O_2_-IC100-IP group: n = 10 mice/group. Scale bars: 50 μm

Next, we tested the inhibitory effects of IC100, a humanized monoclonal anti-ASC antibody in the OIR mouse model. It has been shown that IC100 effectively alleviates symptoms in an animal model of multiple sclerosis induced by chronic inflammation [27]. We administered IC100 immediately after the 5 day 75% O_2_ exposure by multiple IP injections on P12, P14 and P16 or by single IVT injection on P12. IC100 in doses of either 10 or 20 µg/g of mouse body weight was delivered by IP injection in some of the mice, and other mice received IC100 at one dose of 2.5 µg/0.5 µL/eye by IVT injection. PBS as placebo was injected by IVT. AF-594-labeled IB4 stained whole mount retinas showed that IC100 treatment by both IVT and IP (10 μg/g) injections significantly ameliorated vaso-obliteration, intravitreal neovascularization, and related tufts in the wildtype OIR mice compared to placebo-treated OIR controls (Fig. 4d-f). There was no difference in vascular protection between IP treatment with 10 µg/g and 20 µg/g (data not shown). There was no difference in vascular growth between RA-PBS and RA-IC100-IVT treatments (Data not shown). Thus, treatment with IC100 improved vascular development in OIR mice.

### IC100 decreases microglial activation and ASC speck formation in OIR mouse retinas

**Fig. 5:**
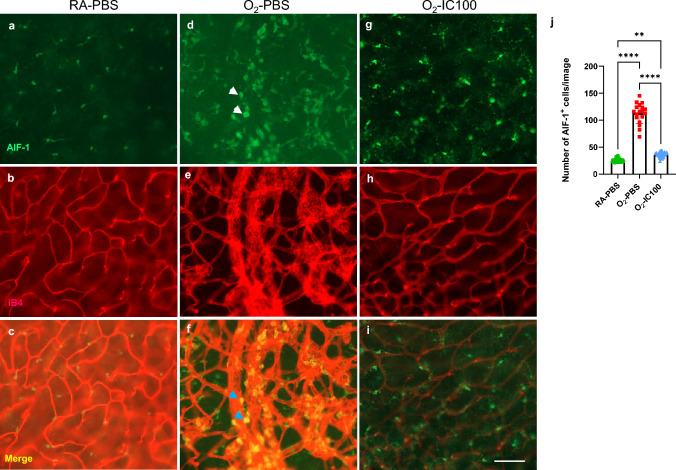
IC100 decreased microglial density and activation in OIR mice. **a**, **d** & **g** Images of retinal whole mount stained by AIF-1 (microglia, green). The microglia in RA-PBS retinas were small and organized (**a**). The microglia in O_2_-PBS retinas had large bodies and increased numbers compared to RA-PBS groups (**d,** white arrow). Treatment with IC100-IVT reduced the number of microglia in O_2_-exposed retinas that appear to have similar shapes as the RA-PBS group (**g)**. **b**, **e** & **h** IB4 stained vasculature (red). The vasculature was normal in the RA-PBS group (**b**). The vasculature was abnormal with increased density and thickness in the O_2_-PBS group (**e**). Treatment with IC100-IVT improved vascular growth in O_2_-exposed retinas compared to the O_2_-PBS group (**h**). **c**, **f** & **i** Merged images. The retinas from O_2_-PBS group had abnormal microglia colocalized with the abnormal vasculature (**f**, blue arrows). **j** Quantification of microglia. The number of microglia in the O_2_-PBS retinas increased more than 70% compared to RA-PBS. While treatment with IC100-IVT drastically reduced the microglial counts by 75% compared to the O_2_-PBS group. **P < 0.01, ****P < 0.0001. n = 4–10 mice/group. Scale bars: 25 µm

Previous studies by other groups and our laboratory reported that microglia activation is induced in OIR mouse models [26, 37]. The numbers of microglia and their activation are highly correlated with inflammation and ROS. To assess whether IC100 reduces microglial cell activation in wildtype OIR mice, we identified microglial cells by immunofluorescence staining of whole mount retinas using an antibody to AIF-1. In the O_2_-PBS group, microglia were more amoeba shaped with an increased nuclear body (Fig. 5d**,** white arrow) compared to RA mouse retinas with a ramified form (Fig. 5a). Activated microglia colocalized with abnormal vessels (Fig. 5f**,** blue arrow), which indicates that microglia activation and inflammation induced by oxygen is highly correlated with abnormal angiogenesis. However, the IC100-IVT treated oxygen-exposed (O_2_-IC100) retinas had microglial cells morphologically similar to those in the RA-PBS group (Fig. 5g). We quantified the numbers of microglia and activated microglia and found they were significantly reduced (75% reduction, Fig. 5j) by IVT treatment with IC100 in OIR retinas compared to placebo-treated (O_2_-PBS) OIR retinas. Take together, these data suggest that IC100 is effective in suppressing microglia activation in OIR mice.

**Fig. 6:**
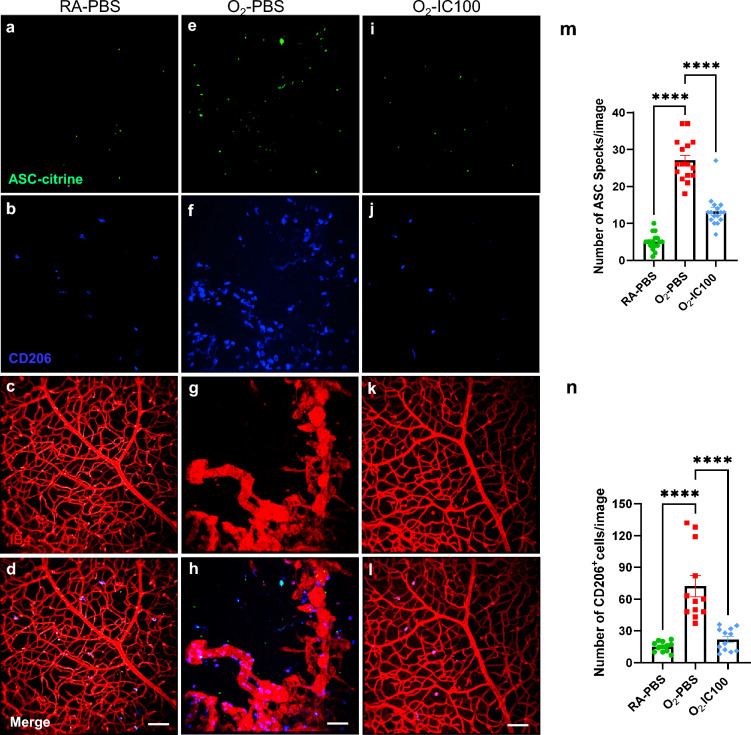
Immunofluorescent confocal microscopy images of OIR model ASC-citrine mouse retinal flat mount staining. **a**–**d** Images of RA-exposed mouse whole mount retinas: **a** ASC-citrine specks (green). **b** CD206 positive microglia (blue). **c** Normal vasculature (red, IB4 staining). **d** Merged image. **e–h **Images of OIR mouse retinal flat mount staining: **e** ASC-citrine specks (Green), **f** CD206 positive microglia (blue), **g** Avascular and neovascular tufts (red), and **h** Merged image. **i**–**l** Images of IC100 treated OIR mouse whole mount retinas: **i** Decreased ASC specks (green), **j** Suppression of CD206 positive microglia (blue), **k** Normal vasculature (red, IB4 staining), and **l** Merged image. **m** Graph of ASC speck quantification. **n** Graph of CD206 positive microglia quantification. n = 3 mice/group, 4 images/retina. ****P < 0.0001

Additionally, we evaluated if IC100 treatment could decrease ASC speck formation and microglia activation in OIR, ASC-citrine reporter mice. Confocal images of retinal flat mounts showed that ASC specks were decreased about 55% by IC100 treatment in the OIR retinas compared to O_2_-PBS retinas (Fig. 6e, i & m). Furthermore, immunofluorescence staining with CD206 demonstrated that IC100 treated OIR mice had 75% decreased CD206 positive microglia compared to O_2_-PBS retinas (Fig. 6f, j & n). The increased ASC specks and CD206 microglia were colocalized with the abnormal vasculature (Fig. 6h**)**. Taken together, these findings suggest that IC100 is effective in suppressing microglia activation by dismantling ASC speck in OIR mouse retinas.

### IC100 attenuates retinal structural pathology in OIR mice

**Fig. 7:**
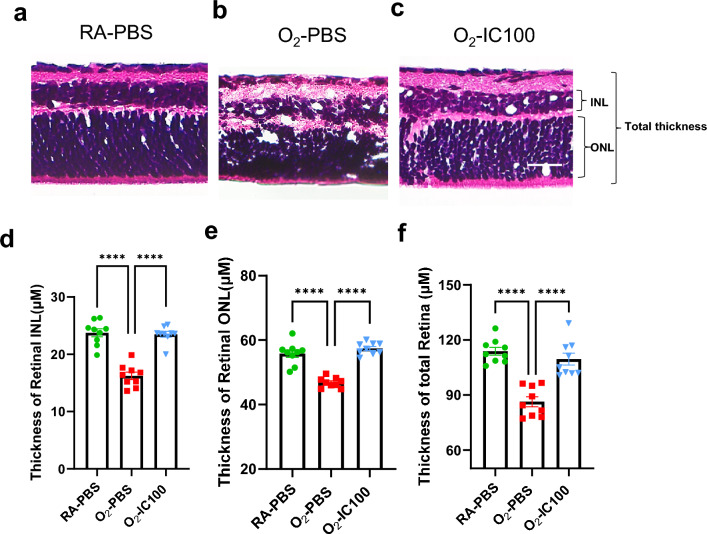
IC100 treatment restored OIR model retinal structure formation. **a**–**c** H&E-stained retinal cross-sections. The O_2_-PBS retinas (**b**) had disorganized GCL, and reduced thickness of the inner nuclear layer (INL), outer nuclear layer (ONL) and total thickness of retinal layer compared to the RA-PBS group (**a**). The O_2_-IC100-IVT group had retinal tissue layer thicknesses that were similar to the RA-PBS group (**c**). **d** Quantification of retinal INL thickness. **e** Quantification of retinal ONL. **f** Quantification of total retinal layer thickness. n = 9 mice. ****P < 0.0001. ns: no significance. Scale bar: 25 µm

We further evaluated retinal histopathology on H&E-stained retinal tissue sections. As illustrated in Fig. 7, the retinal structure in oxygen-exposed and placebo treated mice (O_2_-PBS) had a disorganized GCL with missing ganglion cells. The O_2_-PBS retinas also had reduced thickness of the INL, ONL, and total retina (Fig. 7b). However, treatment with IC100 significantly improved the organization of the GCL as well as the thickness of the INL, ONL, and total retina (Fig. 7c-f). Thus, the reduced ASC activity by IC100 treatment restores retinal structure formation in OIR mouse retinas.

### IC100 restores retinal function in OIR mice

**Fig. 8:**
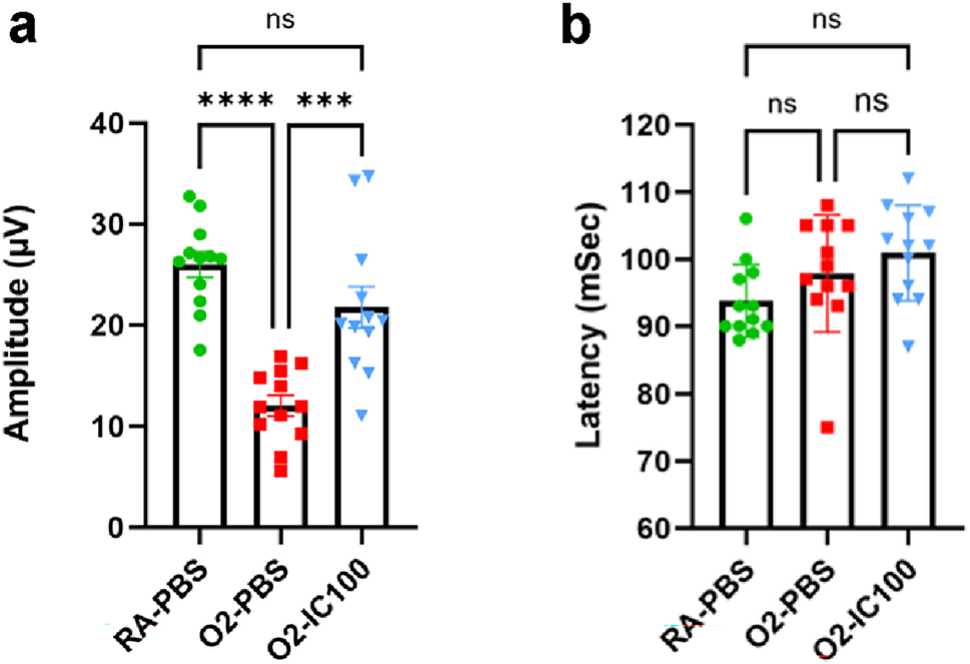
IC100 improves OIR model mouse retinal function. **a** Average amplitude of all mice in each group. The amplitude was reduced to 12.09 µV in the O_2_-PBS retinas compared to 25.99 µV in the RA-PBS group, while treatment with IC100-IVT increased the amplitude to 21.77 µV compared to the O_2_-PBS group. **b** Average latency of all mice in each group. The latency had no significant difference among the three groups group. n = 6 mice/group. ***P < 0.001, ****P < 0.0001, ns: no significance

In addition, we performed PERG to assess whether improved retinal vasculature and tissue layer formation by treatment with IC100 restored OIR retinal function. The amplitude of OIR mice treated with the placebo (O_2_-PBS) had more than 50% reduction compared to the RA control mice (RA-PBS). In comparison, administration of IC100 via IVT injection significantly increased the amplitude by 70% compared to placebo-treated OIR mice (Fig. 8a). The latencies of these 3 groups were of no significant difference (Fig. 8b). Our findings suggest that ASC speck formation triggered by hyperoxia is highly correlated with retinal dysfunction, and that inhibition of ASC by IC100 largely restores retinal function.

### IC100 decreases expression of inflammasome-related molecules, inflammatory cytokines, and VEGF in OIR mouse retinas

**Fig. 9:**
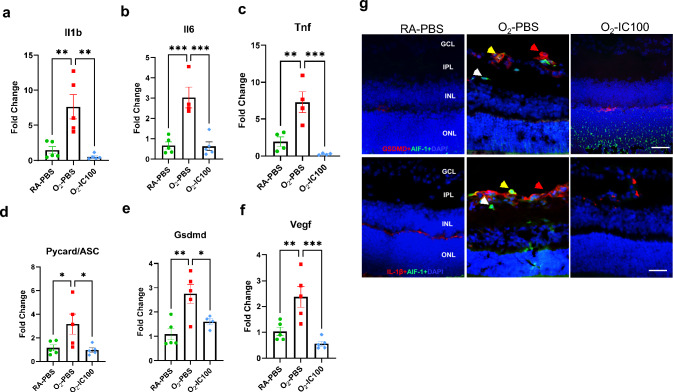
IC100 reduced expression of inflammatory cytokines, inflammasome-related molecules, and Vegf in OIR model mouse retinas. Real-time qRT-PCR of O_2_-PBS retinas showed gene expression of inflammasome cytokines, Il1b, Il6 and Tnf, respectively elevated 7.7, 4.3 and 4.7-fold compared to age-matched RA mouse retinas on P18. These genes were significantly decreased in O_2_-IC100-IVT mouse retinas (**a**–**c**). Pycard/Asc, Gsdmd, and Vegf were increased 2.4 to threefold in O_2_-PBS retinas compared to RA mouse retinas but were significantly decreased in IC100-IVT treated OIR retinas (**d**–**f).** n = 5 mice/group. *P < 0.05, **P < 0.01, ***P < 0.001. Double immunofluorescence staining of anti-GSDMD or anti-IL-1β antibodies (red signal, red arrow) with an anti-AIF-1 antibody (green signal, green arrow) and DAPI staining (blue signal) (**g**). Upper panel: increased GSDMD was detected in AIF-1 positive cells (yellow signal, yellow arrow) in O_2_-PBS retinas, but this was decreased in O_2_-IC100 retinas. Lower panel: increased IL-1β was detected in AIF-1 positive cells (yellow signal, yellow arrow) in O_2_-PBS retinas, however, this was decreased in O_2_-IC100 retinas. Scale bar: 50 μm

To explore the molecular mechanisms by which IC100 protects against OIR in mice, we evaluated expression of inflammasome-related molecules and Vegf in OIR mouse retinas. Real-time qRT-PCR revealed that gene expression of inflammatory cytokines, Il1b, Il6, and Tnf in OIR mouse retinas were respectively increased 7.7, 4.3, and 4.7-fold compared to age-matched RA controls (Fig. 9a-c). Gene expression of Asc, Gsdmd, and Vegf in OIR mouse retinas were significantly increased 3, 2.4, and 2.7-fold compared to age-matched RA controls **(**Fig. 9d-f). However, IC100 treatment significantly decreased gene expression of these inflammasome-related molecules, inflammatory mediators, and Vegf in OIR retinas compared to O_2_-PBS OIR retinas (Fig. 9a-f). There was no statistical difference in gene expression of Nlrp3 and Il18 between RA control and OIR mouse retinas (data not shown). We examined the protein expression of GSDMD and IL-1β, two important targets of inflammasome activation in the retinas. Wildtype mice exposed to hyperoxia and PBS had increased expression of GSDMD and IL-1β in the retinas and some of them was colocalized in AIF-1-stained microglial cells (Fig. 9g). However, treatment with IC100 reduced GSDMD and IL-1β expression in hyperoxia-exposed retinas (Fig. 9g). Therefore, our findings manifest that ASC speck formation induced by hyperoxia leads to inflammasome activation and dysregulation of VEGF in the retinas of OIR mouse models. However, an anti-ASC antibody, IC100 significantly downregulated the expression of these factors.

#### IC100 prevents hyperoxia modulation of gene pathways related to eye development, retinal angiogenesis, VEGF signaling, and leucocyte functions

**Fig. 10:**
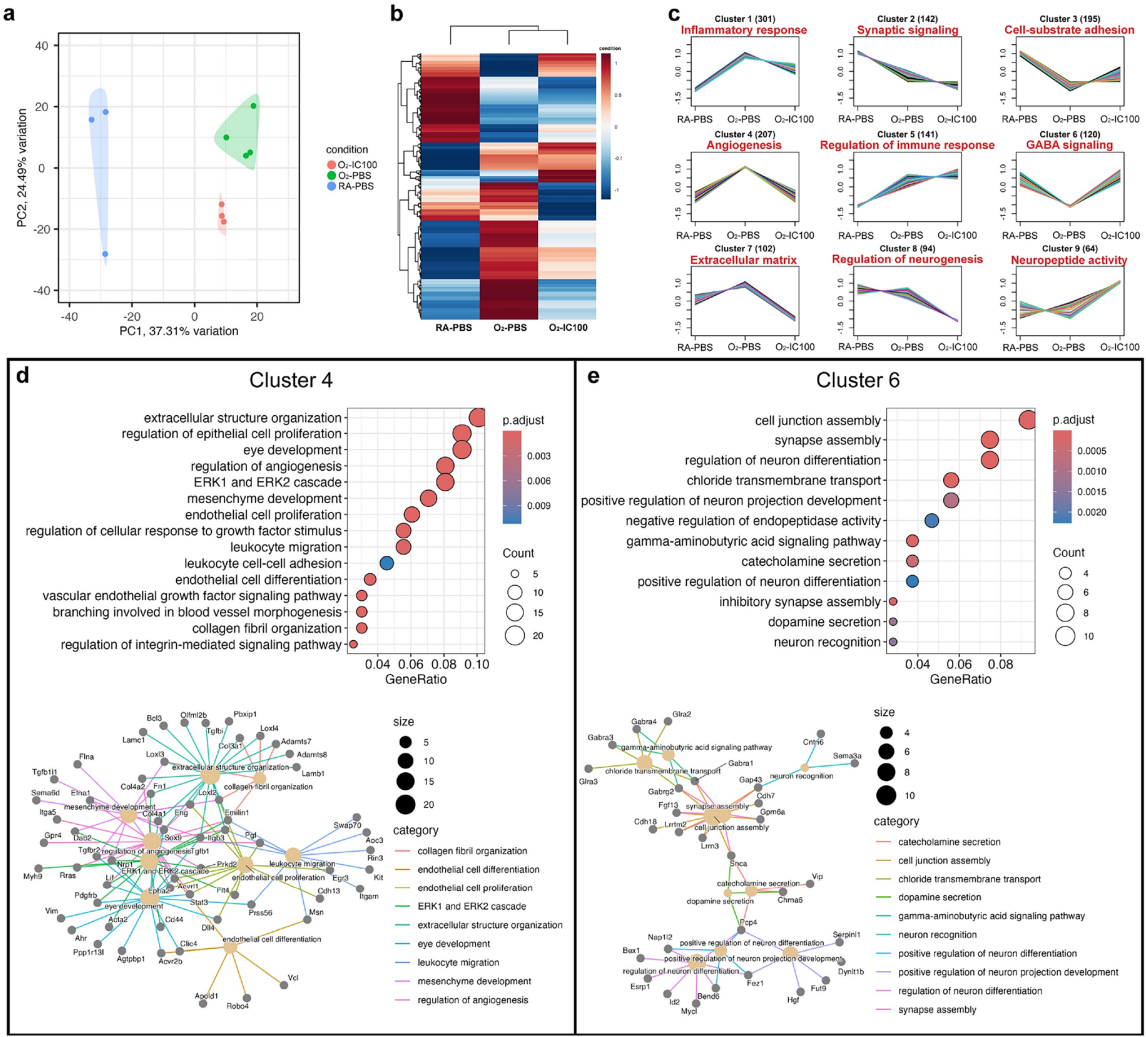
Differential expression and clustering analyses show IC100 prevents hyperoxia modulation of gene pathways related to eye development, retinal angiogenesis, VEGF signaling, and leucocyte functions. **a** Principal component analysis (PCA) plot showing separation of RA-PBS, O_2_-PBS, and O_2_-IC100 by PC1 and PC2. **b** Heatmap of differentially expressed genes showing relative expression. **c** Expression patterns identified in the 9 clusters with the number of genes displayed next to the cluster numbers. **d** Gene pathways upregulated by hyperoxia but corrected by IC100 treatment to the near RA levels in cluster 4 include eye development, angiogenesis, leukocyte migration and cell–cell adhesion, endothelial cell differentiation, and VEGF signaling. **e** Gene pathways that were downregulated by hyperoxia but corrected by IC100 treatment to near RA levels in cluster 6 include cell junction assembly, synapse assembly, and neuron differentiation. n = 3–4 retinas/group

To understand the global transcriptional effects of IC100 treatment that drive the improvement of retinal structure and function under hyperoxia, we performed RNA-seq of retinas from RA-PBS, O_2_-PBS, and O_2_-IC100 groups. The principal component analysis (PCA) plot showed a clear separation of RA-PBS, O_2_-PBS, and O_2_-IC100 by PC1 and PC2, indicating distinct transcriptional profiles in each group (Fig. 10a). We performed differential expression analysis (Fig. 10b) and clustering of differentially expressed genes based on gene expression patterns (Fig. 10c). We identified 9 unique clusters and, with gene set enrichment analysis, identified the main biological process themes for each cluster based on the number of associated genes and adjusted *P* values. These clusters were related to inflammatory response (Cluster 1), synaptic signaling (Cluster 2), cell-substrate adhesion (Cluster 3), angiogenesis (Cluster 4), regulation of immune response (Cluster 5), GABA signaling (Cluster 6), extracellular matrix (Cluster 7), regulation of neurogenesis (Cluster 8), and neuropeptide activity (Cluster 9). Among these clusters, Cluster 4 and Cluster 6 comprised genes in which IC100 treatment corrected their expression in hyperoxia to levels similar to room air. Cluster 4 genes showed striking upregulation in hyperoxia and correction with IC100 treatment (Fig. 10d). Genes set enrichment analysis revealed that genes in this cluster are enriched for eye development, angiogenesis, leukocyte function, and VEGF signaling. Cluster 6 genes showed striking downregulation in hyperoxia and correction with IC100 treatment (Fig. 10e). Genes set enrichment analysis showed that genes in this cluster are enriched for synapse assembly, neuron projection, and neuron differentiation. These results suggest that IC100 corrected hyperoxia-induced abnormal signaling pathways regulating vascular development, inflammatory response, and neuron development.

In order to identify the specific transcriptional effects of hyoperoxia and IC100 treatment, we further performed pairwise differential gene expression analyses between RA-PBS and O_2_-PBS, O_2_-PBS and O_2_-IC100, and RA-PBS and O_2_-IC100 groups. Compared to RA-PBS, 580 genes were induced and 342 genes were suppressed in O_2_-PBS retinas (**Supplemental **Fig. 1a & 1b). Hyperoxia-induced genes were associated with extracellular matrix organization, angiogenesis, inflammatory response, and neuron death (**Supplemental **Fig. 1c & 1e). Hyperoxia-suppressed genes were associated with eye development, neural precursor proliferation, and synaptic transmission (**Supplemental **Fig. 1d & 1f). Compared to O_2_-PBS, 350 genes were induced, and 128 genes were suppressed in the O_2_-IC100 retinas (**Supplemental **Fig. 2a & 2b). IC100-induced gene pathways were associated with extracellular matrix organization, angiogenesis, and tissue remodeling in O_2_-exposed retinas (**Supplemental **Fig. 2c & 2e**).** IC100-suppressed gene pathways in O_2_-exposed retinas were associated with neurogenesis, synapse assembly, and GABA signaling (**Supplemental **Fig. 2d & 2f). Compared to RA-PBS, IC100 upregulated 248 genes and downregulated 284 genes in O_2_-exposed retinas (**Supplemental **Fig. 3a & 3b). IC100 induced gene pathways regulating inflammatory response, angiogenesis, and cell migration (**Supplemental **Fig. 3c & 3e**)**. The gene pathways that were downregulated by IC100 regulate neurogenesis, axonogenesis, and dendrite development (**Supplemental **Fig. 3d & 3f).

## Discussion

In this study, we found that hyperoxia exposure induced ASC specks in the mouse retinas. In an OIR mouse model, we showed that inhibition of ASC speck formation by IC100, a humanized monoclonal anti-ASC antibody, by IVT or IP injection improved vaso-obliteration and intravitreal neovascularization in OIR retinas. These vascular changes were associated with reduced microglial activation, restored retinal structure, and improved retinal function. These structural and functional improvements by IC100 correlated with corrections of hyperoxia-modulated gene pathways associated with eye development, angiogenesis, inflammatory response, and neurogenesis. To the best of our knowledge, this study is the first to demonstrate the efficacy of IC100 in successfully treating OIR mice. Given that IC100 is a humanized antibody, and it is being manufactured for possible use in clinical trials, it may have a potential therapeutic use in the treatment of preterm infants with ROP.

ROP, characterized by avascular retina and intravitreal neovascularization, continues to be a major cause of childhood vision impairment and blindness [1, 4] and current therapies using laser and VEGF inhibitors have unwanted side effects [6–9]. Anti-VEGF therapy by IVT injection is an effective therapy with several advantages compared with laser therapy, including the potential for increased visual field and lower degrees of refractive error [7]. However, there is a paucity of safety data about the effects on organ systems beyond the eye. IVT bevacizumab lowers serum VEGF levels for up to 8 weeks [38], and thus may inhibit vascular development in rapidly developing organs such as the lungs and brain of neonatal infants. In fact, anti-VEGF has been reported to be associated with lower motor scores and higher rates of neurodevelopmental disability in children [39, 40]. Thus, novel, safe, and effective therapies are needed for treating ROP.

Inflammasomes are multi-protein complexes that mediate proteolytic cleavage of GSDMD, pro-IL-1β, and pro-IL-18 by caspase-1 [18]. ASC is pivotal in inflammasome assembly and activation of caspase-1, which subsequently activates GSDMD that leads to pyroptosis, and fast release of IL-1β and IL-18 that results in inflammation [21]. There is increasing evidence that inflammasome activation plays a role in ROP. NLRP3 inflammasome activation is linked to OIR in mice [41], and treatment with MCC950, an inhibitor of NLRP3, reduced the pathological neovascularization in OIR mice [25]. We recently published a study showing that GSDMD gene knockout ameliorated hyperoxia-induced retinal injury including, vaso-obliteration and intravitreal neovascularization, retinal inflammation, retinal layer thinning, and transcriptional regulation of retinal gene pathways related to inflammation, cell death, tissue remodeling, and vascular development [26]. However, there is no report on whether ASC plays a role in OIR or if inhibition of ASC reduces retinal damage in OIR mice.

Herein, we tested if hyperoxia activates ASC in the developing vasculature and retinas. ASC speck formation is widely used as a readout for detecting inflammasome activation [42]. We utilized ASC-citrine reporter mice to examine if hyperoxia activates ASC speck formation in the retinas. We first exposed these mice to RA and 85% O_2_ from P1 to P14, which commonly used to induce BPD and multi-organ injuries in neonatal mice [26, 36]. We found that ASC specks were increased in hyperoxia-exposed lungs and brains in these mice (data not shown). When we examined the retinas, we found no ASC specks in RA-exposed mice. However, the hyperoxia-exposed retinas had increased ASC specks associated with disorganized vasculature. We also found a similar increase in retinal ASC specks and vascular association when neonatal ASC-citrine reporter mice and wildtype mice were exposed to 75% O_2_ from P7 to P11, which is commonly used in mice to induce ROP-like OIR models. Moreover, ASC specks were colocalized with both M1 and M2 activated microglia in the intravitreal neovascular areas of the OIR retinas. Additional studies with the OIR model in wildtype mice also detected ASC specks in retinal ganglion cells and microglia cells, as well as the vitreous fluid of the OIR mice. These novel data highlight that ASC is activated in the retinas of OIR mice, which sets a foundation for us further test the efficacy of ASC inhibition in treating OIR mice.

We tested IC100 for treatment of mouse OIR by IVT injection at P12 and IP injection at P12, P14, and P16 during the post hyperoxia exposure and room air recovery period. We performed detailed assessments of intravitreal vascularization, microglial expression, retinal layer thickness, retinal function, and transcriptome profiling. We have demonstrated that IC100 effectively treated both phases of OIR as IC100-IVT and IC100-IP had decreased retinal vaso-obliteration and intravitreal vascularization, with IC100-IVT being more effective. Similar to our recent publication on the role of GSDMD in OIR, we found that IC100 treatment reduced hyperoxia-induced retinal inflammation as assessed by reduced numbers of activated microglial cells detected by AIF-1 (M1 microglia/macrophages) and CD206 (M2 microglia/macrophages) in the retinas. Similar findings of M1 and M2 microglial cells both existing in OIR mouse retinas have been previously reported [33]. Microglial cells play active roles in maintaining the normal structure and functioning of the retina under normal physiological conditions. Microglia become pathologically activated in a chronic pro-inflammatory environment and release excessive inflammatory mediators that promote retina damage and disease progression [43–45]. Preventing chronic microglial activation by IC100 would certainly reduce retina injury and the progression of OIR caused by chronic hyperoxia exposure.

Previous studies have demonstrated that mouse OIR models have reduced retina thickness in multiple layers [26, 46]. We sought to understand the role of ASC in retinal structure development by analyzing retinal thickness. We found that under hyperoxia exposure, the placebo-treated mice had retinal thinning compared to the IC100-IVT and IC100-IP-treated hyperoxia-exposed retinas, with major differences observed in the INL, ONL and total retinal thickness. We also observed disorganized GCL with missing ganglion cells in oxygen-exposed, placebo-treated retinas. However, treatment with IC100 significantly reduced the thinning of these layers and improved the organization of the GCL. The INL has second order neuron bipolar cells, and the ONL has cone photoreceptors [47]. Bipolar cells separate visual signals evoked by light and dark contrasts and encode them to ON and OFF pathways, respectively [48]. Photoreceptors are specialized neurons that convert light into electrical signals that stimulate physiological processes. Signals from the photoreceptors are sent through the optic nerve to the brain for processing [49]. Thus, any therapeutic that reduces thinning of these tissue layers and improves GCL organization as we have shown that IC100 does, should help maintain or restore retina structural development and improve ROP functional outcomes.

To that end, we tested if decreased pathological vascularization, reduced inflammation, and restored retinal layer development by IC100 treatment led to improved retinal function as assessed by PERG in our OIR model. We found that placebo injected hyperoxia-exposed retinas had drastically decreased amplitude, however, treatment with IC100 by IVT injection significantly increased amplitude in the hyperoxia-exposed mice. Previous studies in OIR mice have shown that this model not only has abnormal vasculature, but also significantly reduced neuronal function [30, 50]. In a study similar to ours it was recently reported that a humanized monoclonal antibody Fab fragment to the angiogenic factor secretogranin III, effectively corrected retinal dysfunction in OIR mice [30]. These data suggest an potential role for antibodies against both inflammasome-related molecules and angiogenic factors in regulating retinal functions under hyperoxia that result in ROP.

Our study provided molecular insights into the underlying mechanisms by which IC100 treatment improves retinal structure and function in this OIR model. We showed by qRT-PCR that IC100 specifically downregulated hyperoxia-induced gene expression of inflammasome-related molecules such as Asc, Gsdmd, and Il1b, inflammatory mediators, Il6 and Tnf, and an angiogenic factor, Vegf. Our retinal RNA-seq data provided a better characterization of how IC100 affects hyperoxia-regulated transcriptomes and biological pathways related to OIR. We found that hyperoxia upregulated and downregulated distinctive gene pathways in the PBS-treated retinas but IC100 treatment corrected some of these gene pathways under hyperoxia. In O_2_-PBS retinas, hyperoxia-induced gene pathways in cluster 4 were corrected by IC100 to about room air levels, including extracellular structure organization, mesenchyme development, regulation of angiogenesis, regulation of eye development, endothelial cell differentiation and proliferation, and leukocyte migration. Some specific genes in these pathways included TGF beta induced (Tgfbi) [51], collagen type III alpha 1 chain (Col3a1) [52], lysyl oxidase like-4 (Loxl4) [53], and fibronectin (Fn1) [54] (extracellular structure organization); Loxl2 and Loxl3 [53], and integrin subunit beta 3 (Itgb3) [55] (mesenchyme development); Sox9 [56], placenta growth factor (Pigf) [57], and G protein-couple receptor 4 (Gpr4) [58] (angiogenesis); vimentin (Vim) [59], aryl hydrocarbon receptor (Ahr) [60], chloride intracellular channel 4 (Clic 4) [61], and ATP/GTP binding protein 1 (Agtpbp1) [62] (eye development); and putative serine protease 56 (Prss56) [63], moesin (Msn) [64], integrin alpha M (Itgam) [65], amine oxidase copper containing 3 (Aoc3) [66], and switching B cell complex subunit (Swap70) [67] (leukocyte migration). Many of these genes have been associated with adult retinal diseases. Our data are the first to show that these genes could be direct or indirect target genes of the inflammasome cascade, which play important roles in OIR. We also identified some of the hyperoxia-suppressed gene pathways that were corrected by IC100 in cluster 6 including synapse assembly, regulation of neuron differentiation, projection, and recognition. Some of specific genes in these pathways included fibroblast growth factor 13 (Fgf13) [68], cadherin 18 (Cdh18) [69], and growth associated protein 43 (Gap43) [70] (synapse assembly); nucleosome assembly protein 1-like 2 (Nap1l2) [71], fasciculation and elongation protein zeta-1 (Fez1) [72], BEN domain containing 6 (Bend6) [73], serpine family I member 1 (Serpini1) [74], alpha-(1,3)-fucosyltransferase (Fut9) [75], and hepatocyte growth factor (Hgf) [76] (regulation of neuron differentiation and projection development). The known functions of some of these genes are related to photoreceptor degeneration, retinal degeneration, retinal development, neural circuit establishment, and self-renewal of neural stem cells. Their roles in the development of OIR or ROP have not been established. The fact that IC100 treatment improved retinal structure and function and corrected expression of these genes further supports the critical role of ASC in the pathogenesis of OIR by transcriptional regulation of hyperoxia-modified genes in mouse models.

We recognize that treatment with IC100 partially corrected the transcriptional changes induced by hyperoxia compared to normal-developed retinas in the RA-PBS group. These results are expected, given that IC100 treatment was provided on P12 when the retinas had been exposed to hyperoxia for 5 days, and the vascular injury and inflammation were induced by hyperoxia at this time. This hyperoxia exposure and treatment sequence closely resemble the clinical practice in ROP diagnosis and treatment.

In conclusion, this study demonstrates that hyperoxia activates ASC in mouse retinas. Treatment with IC100 largely attenuates abnormal vascularization, inflammation, retinal thinning, and retinal dysfunction in a mouse model of OIR. We provide transcriptional effects of hyperoxia and IC100 treatment in addition to the structural and functional changes in this model. The results from this study, combined with our recently published data on the effects of GSDMD deficiency in ameliorating hyperoxia-induced lung and retinal injury in neonatal mice [26], highlight that the inflammasome-cascade is central to hyperoxia-induced premature multi-organ damage including the retinas. Additional studies are needed to test this antibody in other rodent models of OIR to gain evidence that targeting ASC may be beneficial for treating ROP in premature infants.

### Supplementary Information

Below is the link to the electronic supplementary material.

Supplementary file1 (PDF 185 kb)

Supplementary file3 (PDF 2366 kb)

## Data Availability

The datasets generated during the current study are available in GEO@ncbi.nlm.nih.gov under record GSE261490.
