# Supplementary material for: IC100, a humanized therapeutic monoclonal anti-ASC antibody alleviates oxygen-induced retinopathy in mice
Source: Angiogenesis. 2024 May 6;27(3):423–40. doi: 10.1007/s10456-024-09917-9 (PMC11303442; doi:10.1007/s10456-024-09917-9)
Supplement: Supplementary file 1 — Supplementary file1 (PDF 185 kb) [file 10456_2024_9917_MOESM1_ESM.pdf]

Online Supplement - Materials

**Antibody list**	**Company**	**Item number**	**Usage**
Goat anti-AIF-1	NovusBio	NB100-0128	Immunofluorescence (1:200)
Mouse Anti-ASC	Santa Cruz	sc-515414	Immunofluorescence (5.0 mg/ml)
Mouse Anti-GSDMDC1 (A7)	Santa Cruz	sc-393656	Immunofluorescence (1:50)
Mouse Anti-IL-1β (3A6)	Cell signaling	12242	Immunofluorescence (1:200)
Alexa Fluor-594-isolection B4	ThermoFisher	I21413	Immunofluorescence (20 µg/ml)
Brilliant Violet 421™Anti-mouse CD206	BioLegend®	141717	Immunofluorescence (1:250)
Goat anti-Mouse IgG (H+L) Secondary Antibody, HRP	ThermoFisher	31430	Western blot (1:10,000)
Donkey anti-Mouse IgG (H+L) ReadyProbes™ Secondary Antibody, Alexa Fluor™ 594	ThermoFisher	R37115	Immunofluorescence (1:200)
Donkey anti-Goat IgG (H+L) Cross-Adsorbed Secondary Antibody, Alexa Fluor™ 594	ThermoFisher	A-11058	Immunofluorescence (1:200)
**PCR primer list**	**Company**	**Item number**	**Usage**
Il1b	ThermoFisher	Mm00434228_m1	qRT-PCR
Il6	ThermoFishe	Mm00434228_m1	qRT-PCR
Il18	ThermoFisher	Mm00434226_m1	qRT-PCR
Tnf	ThermoFisher	Mm00443258_m1	qRT-PCR
Asc	ThermoFisher	Mm00445747_g1	qRT-PCR
Gsdmd	ThermoFisher	Mm00509958_m1	qRT-PCR
Nlrp3	ThermoFisher	Mm00443258_m1	qRT-PCR
Vegf	ThermoFisher	Mm01281449_m1	qRT-PCR
**Other reagents**	**Company**	**Item number**	**Usage**
miRNeasy Mini Kit	Qiagen	217004	RNA isolation
SuperScript™ III CellsDirect™ cDNA Synthesis Kit	ThermoFisher	18080200	cDNA synthesis
cOmplete ULTRA Tablets, Mini, *EASYpack,* Protease Inhibitor Cocktail	Roche	05892970001	Western blot

Online Supplement – Results

Figure 1. Hyperoxia dysregulates gene pathways associated with tissue remodeling, angiogenesis, inflammation, and eye development. a. Volcano plot of differentially expressed genes between O_2-PBS and RA-PBS (red: FDR<0.1 and fold-change > 1.5). **b.** Heatmap of differentially expressed genes between O_2-PBS and RA-PBS. Hyperoxia induced 580 genes and suppressed 342 genes. **c.** Dot plot showing gene set enrichment analysis for Gene Ontology terms associated with genes induced by hyperoxia. Hyperoxia-induced genes were associated with extracellular matrix organization, angiogenesis, and inflammatory response. **d.** Dot plot showing gene set enrichment analysis for Gene Ontology terms associated with genes suppressed by hyperoxia**.** Hyperoxia-suppressed genes were associated with eye development, neural precursor proliferation, and synaptic transmission. **e.** Network plot for Gene Ontology Biological Processes associated with genes induced by hyperoxia compared to room air. **f.** Network plot for Gene Ontology Biological Processes associated with genes suppressed by hyperoxia compared to room air.

Figure 2. IC100 modulates hyperoxia-induced and suppressed gene pathways in hyperoxia-exposed retinas. a. Volcano plot of differentially expressed genes between O_2-IC100 and O2-PBS (red: FDR<0.1 and fold-change > 1.5). **b.** Heatmap of differentially expressed genes between O_2-IC100 and O_2-PBS. Hyperoxia induced 350 genes and suppressed 128 genes. **c.** Dot plot showing gene set enrichment analysis for Gene Ontology terms associated with genes induced by IC100 in the setting of hyperoxia. IC100-induced genes were associated with extracellular matrix organization, angiogenesis, and tissue remodeling. **d.** Dot plot showing gene set enrichment analysis for Gene Ontology terms associated with genes suppressed by IC100 in hyperoxia. IC100

suppressed genes associated with neurogenesis, synapse assembly, and GABA signaling. **e.** Network plot for Gene Ontology Biological Processes associated with genes induced by IC100 in the setting of hyperoxia compared to hyperoxia alone. **f.** Network plot for Gene Ontology Biological Processes associated with genes suppressed by IC100 in the setting of hyperoxia compared to hyperoxia alone.

Figure 3. Comparison of gene pathways in RA-PBS and O_2-IC100 retinas. a. Volcano plot of differentially expressed genes between O_2-IC100 and RA-PBS (red: FDR<0.1 and fold-change > 1.5). **b.** Heatmap of differentially expressed genes between O_2-IC100 and RA-PBS. IC100 induced 248 genes and suppressed 284 genes in O_2-exposed retinas compared to normal retinas. **c.** Dot plot showing gene set enrichment analysis for Gene Ontology terms associated with genes induced by IC100 in O_2-exposed retinas compared to normally developing retinas. IC100-induced genes related to inflammatory response, angiogenesis, and cell migration. **d.** Dot plot showing gene set enrichment analysis for Gene Ontology terms associated with genes suppressed by IC100 in O_2-exposed retinas compared to normally developing retinas. IC100 suppressed genes related to neurogenesis, axonogenesis, and dendrite development. **e.** Network plot for Gene Ontology Biological Processes associated with genes induced by IC100 in O_2-exposed retinas compared to RA-exposed retinas. **f.** Network plot for Gene Ontology Biological Processes associated with genes suppressed by IC100 in O_2-exposed retinas compared to RA-exposed retinas.
