# Supplementary figures and images for: IC100, a humanized therapeutic monoclonal anti-ASC antibody alleviates oxygen-induced retinopathy in mice
Source: Angiogenesis. 2024 May 6;27(3):423–40. doi: 10.1007/s10456-024-09917-9 (PMC11303442; doi:10.1007/s10456-024-09917-9)

Figure 1

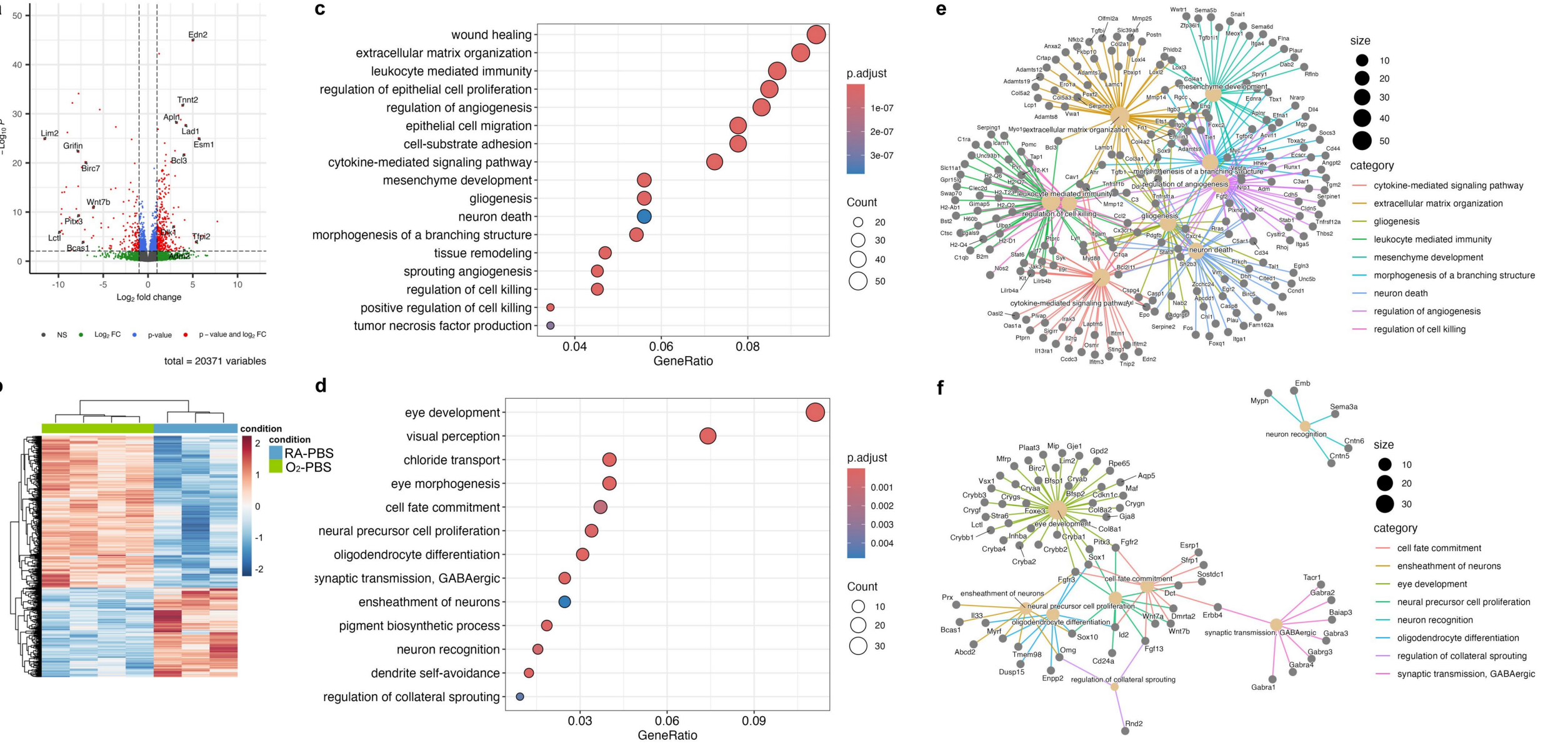

Figure 2

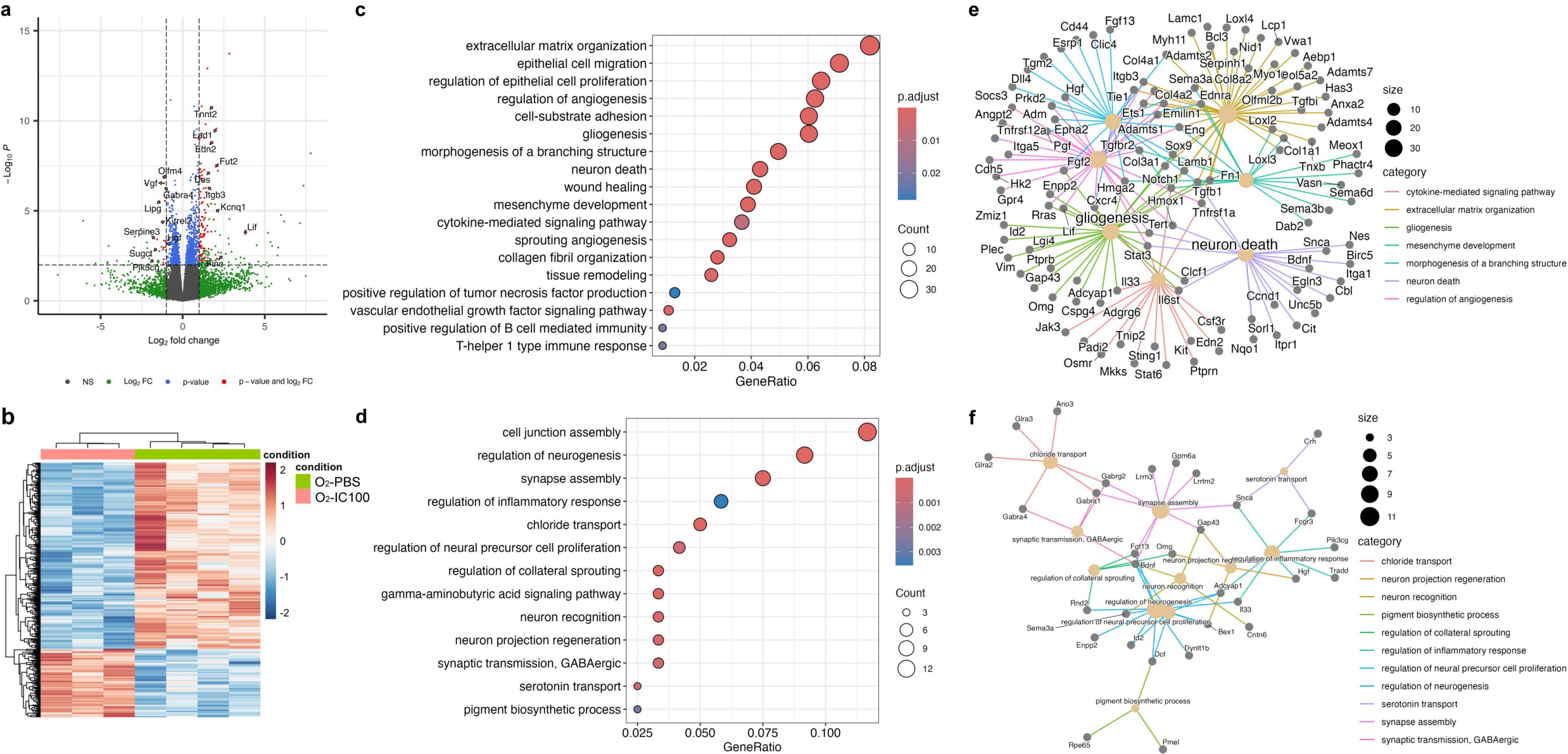

Figure 3

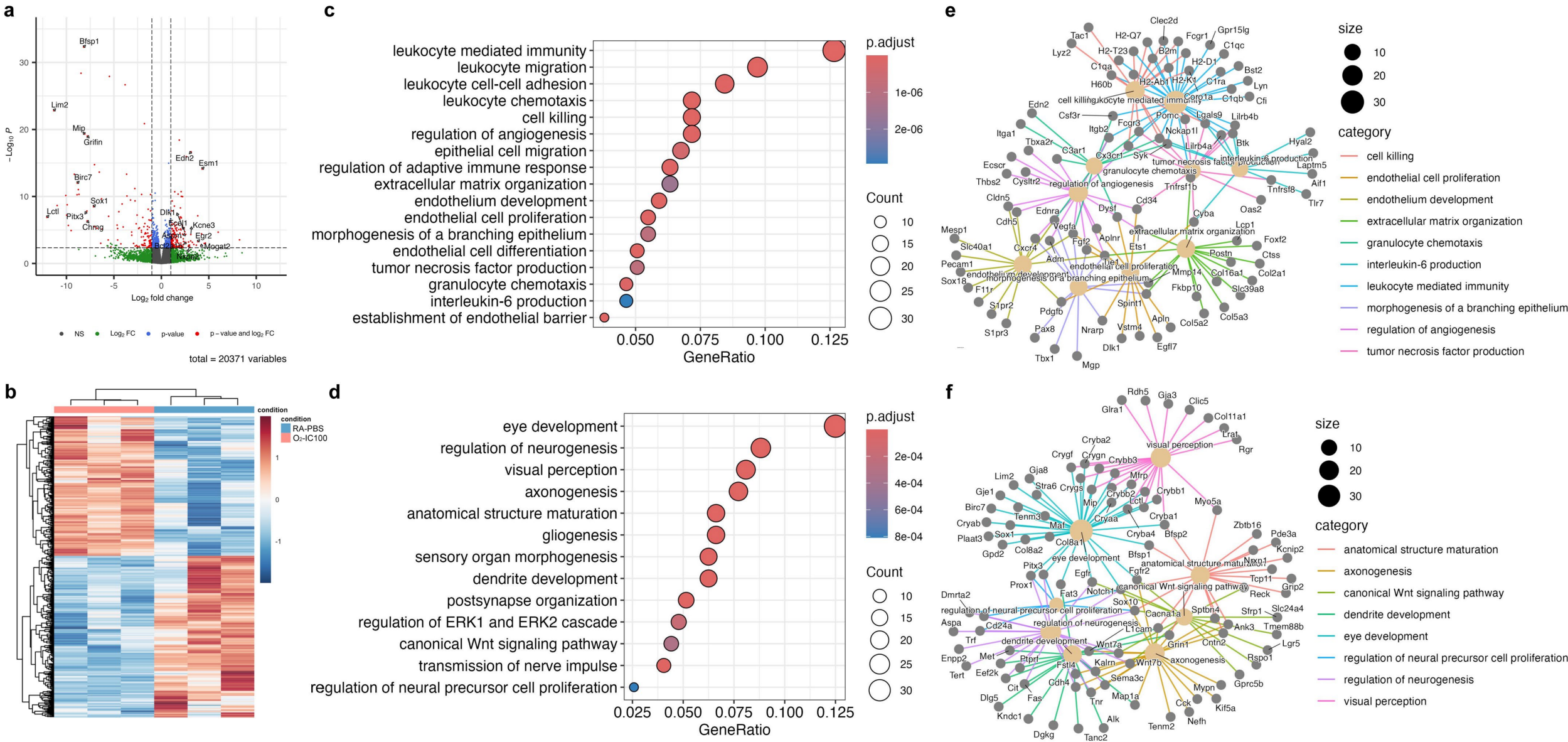

Supplement: Supplementary file 2 — Supplementary file3 (PDF 2366 kb) [file 10456_2024_9917_MOESM2_ESM.pdf]
